# Agomelatine Mitigates Kidney Damage in Obese Insulin-Resistant Rats by Inhibiting Inflammation and Necroptosis via the TNF-α/NF-ĸB/p-RIPK3 Pathway

**DOI:** 10.3390/ijms26051940

**Published:** 2025-02-24

**Authors:** Sasivimon Promsan, Nattavadee Pengrattanachot, Nichakorn Phengpol, Prempree Sutthasupha, La-ongdao Thongnak, Krit Jaikumkao, Anusorn Lungkaphin

**Affiliations:** 1 Department of Physiology, Faculty of Medicine, Chiang Mai University, Intravaroros Road, Chiang Mai 50200, Thailand; sasivimon_pr@cmu.ac.th (S.P.); nattavadee_peng@cmu.ac.th (N.P.); nichakorn_ph@cmu.ac.th (N.P.); prempree_sut@cmu.ac.th (P.S.); 2 Division of Physiology, School of Medical Science, University of Phayao, Phayao 56000, Thailand; 3 Princess Srisavangavadhana College of Medicine, Chulabhorn Royal Academy, Bangkok 10210, Thailand; laongdao.tho@cra.ac.th; 4 Department of Radiologic Technology, Faculty of Associated Medical Sciences, Chiang Mai University, Chiang Mai 50200, Thailand; krit.ja@cmu.ac.th; 5 Functional Foods for Health and Disease, Department of Physiology, Faculty of Medicine, Chiang Mai University, Chiang Mai 50200, Thailand; 6 Functional Food Research Center for Well-Being, Multidisciplinary Research Institute, Chiang Mai University, Chiang Mai 50200, Thailand

**Keywords:** agomelatine, inflammation, insulin resistance, kidney injury, necroptosis, obesity

## Abstract

Obesity is a risk factor for chronic kidney disease. The expansion of adipose tissues in obesity induces insulin resistance and low-grade systemic inflammation, promoting kidney damage. Our previous studies have demonstrated that agomelatine (AGOM) exerts renoprotective effects in experimental models of obesity and insulin resistance through various mechanisms, including the attenuation of ER stress and oxidative stress. This study aimed to further explore the effects of agomelatine on renal inflammation, insulin signaling, and necroptosis in obese, insulin-resistant rats. Obesity was induced in rats with a high-fat diet for 16 weeks, followed by 4 weeks of treatment with 20 mg kg^−1^ day^−1^ of AGOM or 10 mg kg^−1^ day^−1^ of pioglitazone (PIO). The results showed that insulin resistance was improved after treatment with AGOM and PIO, as demonstrated by the reduction in fasting plasma glucose, insulin, and HOMA-IR. Both treatments restored the levels of renal insulin signaling proteins. Moreover, AGOM inhibited TNFα, TNFR1, NF-ĸB, COX2, and IL1β, which attenuated the necroptosis-related proteins RIPK3 and MLKL. AGOM also prevented kidney DNA fragmentation, as detected by the TUNEL assay. In an obese condition, the level of the tight junction protein claudin-1 (CLDN1) was enhanced after being treated with AGOM. In conclusion, the novel mechanisms associated with AGOM and involved in limiting kidney injury were the inhibition of the TNFα/NF-ĸB/p-RIPK3 pathway and a reduction in inflammation and necroptosis. This suggested that AGOM could be an effective treatment for inhibiting kidney dysfunction in cases of obesity and insulin resistance. These findings open new avenues for the management of renal dysfunction, with implications for personalized medicine.

## 1. Introduction

In recent years, obesity rates have risen globally, and uncontrolled food consumption is a major driver. The elevation of free fatty acids (FFAs) in the bloodstream due to obesity is associated with insulin resistance, potentially resulting in the development of type 2 diabetes mellitus (T2DM), cardiovascular disease and chronic kidney disease [1,2]. It has been reported that an increase in adipocyte size leads to cell hypoxia, causing unhealthy adipose tissue. This, in turn, stimulates macrophage infiltration and the release of pro-inflammatory cytokines including monocyte chemotactic protein 1 (MCP1), tumor necrosis factor-α (TNF-α), interleukin 6 (IL-6), and interleukin 1 (IL-1) [3]. These have the potential to induce low-grade systemic inflammation, promoting renal cell inflammation and renal injury [2].

The prolonged consumption of a high-fat diet and fat accumulation aggravate kidney damage and kidney dysfunction, which have been mainly associated with renal oxidative stress, apoptosis, ER stress and inflammation [4,5,6,7]. These can stimulate the release of TNF-α from unhealthy adipose tissue, which induces low-grade inflammation and adversely affects kidney tissue when it binds with TNF receptors (TNFR). This subsequently leads to the activation of the transcription factor nuclear factor kappa B (NF-κB), increases the release of pro-inflammatory cytokines and induces kidney cell inflammation and damage [8,9].

Necroptosis is a form of programmed cell death that mimics the features of apoptosis and necrosis, which are mediated by receptor-interacting serine/threonine kinase 3 (RIPK3) and mixed lineage kinase domain-like protein (MLKL) [10]. This form of programmed cell death responds to the activation of TNFα under pathological conditions. The interaction between TNFα and its receptor, TNFR-1, can activate RIPK1 kinase and subsequently promote the activation of RIPK3 kinase. Then, the activation of RIPK3 kinase triggers the oligomerization of MLKL, which subsequently translocates to the cell membrane, leading to the rapid lysis of the plasma membrane and causing the release of damage-associated molecular patterns (DAMPs) [11]. To date, recent studies have reported that inflammation and the necroptosis pathway cause damage in several organs, in association with various conditions [12].

The increase in FFAs in high-fat-diet-fed rats can activate TLR4 and contribute to the stimulation of NF-kB and the upregulation of RIPK3 in the pancreas, leading to acute pancreatitis [13]. It has also been reported that liver insulin resistance can be observed in obese mice (ob/ob, db/db or diet-induced obesity) through the activation of necroptosis, indicated by the increase in RIPK1, RIPK3 and MLKL levels. Nevertheless, hepatic insulin sensitivity could be improved via the inhibition of MLKL or other necroptosis proteins [14]. Despite these findings, to date, the involvement of necroptosis in kidney pathogenesis in an obese condition is not clearly understood.

The treatment of insulin resistance is currently a crucial goal in the management of T2DM. The medication pioglitazone (PIO) works by improving insulin sensitivity. PIO acts as an agonist for peroxisome proliferator-activated receptor-gamma (PPAR-γ). It has been reported that PIO improves insulin resistance through a reduction in oxidative stress and inflammation, and enhances insulin sensitivity in a T2DM rat model induced by a high-carbohydrate diet [15]. In this study, PIO was used as a positive control.

It has been reported that agomelatine (AGOM), a melatonin analogue, can improve renal function in various kidney injury models. AGOM was shown to reduce kidney inflammation induced by cecal ligation puncture (CLP) via the moderation of TNF-α and pro-inflammatory cytokines [16]. In gentamicin-induced nephrotoxicity in rats, AGOM suppressed the expression of TLR4/NF-kB signaling and reduced TNF-α, IL-1β, nitric oxide (NO) and myeloperoxidase (MPO), which consequently resulted in improved kidney function [17]. Moreover, in obese insulin resistance in rats, AGOM was shown to protect against kidney dysfunction through the inhibition of renal oxidative stress, apoptosis and endoplasmic reticulum (ER) stress [5,6]. This study aimed to further explore the renoprotective effects of agomelatine on renal inflammation, insulin signaling, and necroptosis in obese, insulin-resistant rats.

## 2. Results

### 2.1. Effects of 16 Weeks of High-Fat Diet Feeding on Metabolic Parameters and Renal Function in Rats

At the beginning of the experiment, there were no significant differences in body weight between the groups, as shown in Table 1. After 16 weeks, the body weight was significantly increased in rats fed on a high-fat diet compared to those fed a normal diet (*p* < 0.05). Insulin resistance was observed in the HFD groups (HFD, HFD + AGOM, and HFD + PIO), as indicated by the significant increases in the HOMA-IR and fasting plasma glucose levels in comparison to the ND group (*p* < 0.05). However, there were no significant differences in the plasma insulin levels between the experimental groups. In addition, there were no significant differences in the plasma triglyceride and cholesterol levels between the experimental groups. Impaired renal function was observed in rats fed on a high-fat diet for 16 weeks, as evidenced by a significant increase in serum creatinine compared to the ND group (*p* < 0.05), as seen in our previous study [5]. Taken together, rats fed on a high-fat diet for 16 weeks developed early-stage insulin resistance. This duration of high-fat diet consumption adversely affected kidney function, leading to impaired renal function.

### 2.2. Effects of Agomelatine on Insulin Resistance and Kidney Dysfunction in High-Fat Diet-Induced Obesity in Rats

High-fat diet consumption induced a significant increase in body weight in the HFD group in comparison with ND rats (*p* < 0.05) (Table 2). Kidney weight also tended to rise in the HFD group. On the other hand, the HFD group demonstrated a significant decrease in the kidney weight to body weight ratio (KW/BW) when compared to ND rats (*p* < 0.05). The treatment with AGOM and PIO caused a significant reduction in the body weight and kidney weight in high-fat-treated rats (*p* < 0.05). Obese rats also showed the characteristics of insulin resistance, evidenced by the significant increases in fasting plasma glucose, plasma insulin and HOMA-IR when compared with ND rats (*p* < 0.05). AGOM or PIO treatment resulted in a decrease in these parameters when compared with HFD rats (*p* < 0.05). Dyslipidemia was observed in the HFD group, as demonstrated by a significant increase in plasma cholesterol levels when compared to the ND group (*p* < 0.05). AGOM administration significantly reduced plasma cholesterol levels (*p* < 0.05), whereas there was no statistically significant difference in the plasma cholesterol in the HFD + PIO group compared to obese rats. There were no significant changes in the plasma triglyceride levels among the experimental groups. Kidney dysfunction was observed in the HFD group, as demonstrated by the increase in serum creatinine and urine protein levels. This alteration was significantly attenuated in the AGOM and PIO treated group in comparison with the HFD group (*p* < 0.05). Taken together, AGOM administration could improve the obese and insulin-resistant condition. AGOM and PIO provided the same effects when preventing the impairment of renal function.

### 2.3. Effects of Agomelatine on Visceral Fat Mass and Kidney Lipid Synthesis in High-Fat Diet-Induced Obesity in Rats

**Figure 1:**
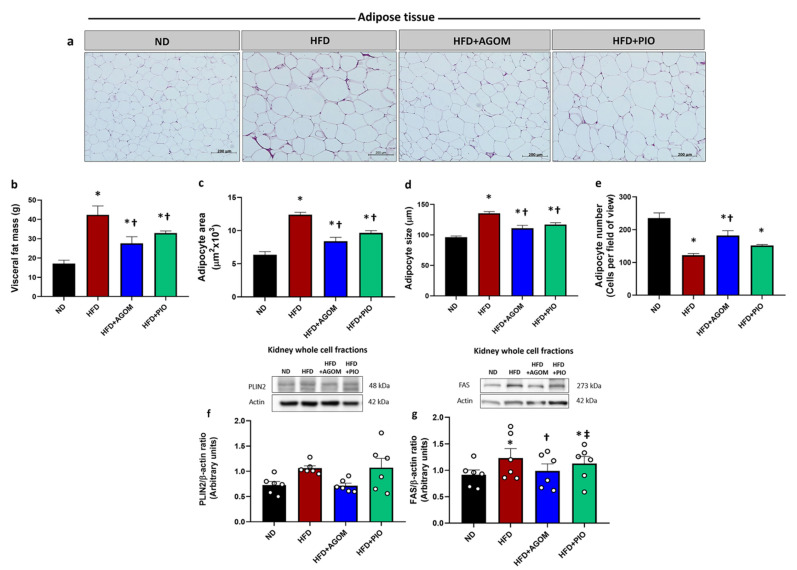
The effects of agomelatine on adipose tissue parameters in visceral fat in high-fat-diet-induced insulin-resistant rats. Hematoxylin and Eosin (H&E) staining of visceral fat tissue (x20) (**a**), visceral fat mass (**b**), adipocyte area (**c**), adipocyte size (**d**) and adipocyte number (**e**). Representative Western blot and the quantitative expression of lipid droplet markers in kidney tissues normalized to β-actin; perilipin 2 (PLIN2) (**f**) and fatty acid synthase (FAS) (**g**). Bar graphs show mean ± standard error of mean (SEM), *n* = 5–6 rats per group. ND: normal-diet-fed rats; HFD: high-fat-diet rats; HFD + AGOM: high-fat diet treated with agomelatine at a dose of 20 mg kg^−1^ day^−1^; HFD + PIO: high-fat diet treated with pioglitazone at a dose of 10 mg kg^−1^ day^−1^. * *p* < 0.05 vs. ND; † *p* < 0.05 vs. HFD; ‡ *p* < 0.05 vs. AGOM.

The H&E staining of adipose tissue is shown in Figure 1a. HFD rats showed an increase in visceral fat mass (Figure 1b) compared to ND rats (*p* < 0.05). In addition, both the adipocyte area (Figure 1c) and adipocyte size (Figure 1d) were significantly higher in the HFD rats than those in the ND controls (*p* < 0.05). In contrast, the adipocyte number (cells per field of view) was significantly reduced in the HFD group when compared with ND rats (*p* < 0.05) (Figure 1e). PLIN2 expression, a marker of lipid accumulation, tended to increase in the kidney tissue of the HFD group. Treatment with AGOM had a tendency to reduce PLIN2, while PIO did not result in a significantly different level of PLIN2 when compared with HFD rats (Figure 1f).

The expression of fatty acid synthase (FAS), a rate-limiting enzyme in the fatty acid synthesis pathway, was significantly elevated in the kidney tissue of HFD rats in comparison to those in the ND group (*p* < 0.05). This was markedly attenuated after treatment with AGOM (*p* < 0.05) (Figure 1g). However, PIO had no effect on the expression of FAS when compared with the HFD controls. The expression of FAS in the HFD + PIO group was higher than that in the HFD + AGOM rats (*p* < 0.05). These results indicated that AGOM reduced fat accumulation in adipose tissue and the adipocyte size. Moreover, it slightly decreased fat accumulation and reduced the level of fatty acid synthase in the kidney tissue under obese conditions.

### 2.4. Effects of Agomelatine on Kidney Inflammation in High-Fat Diet-Induced Obesity in Rats

**Figure 2:**
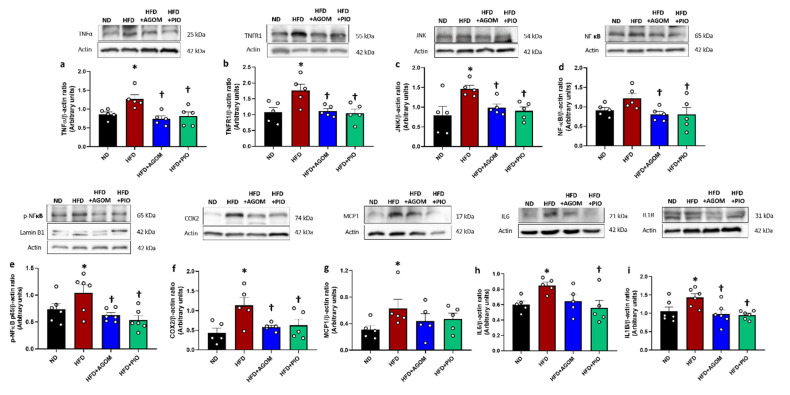
The effects of agomelatine on renal inflammatory proteins in high-fat-diet-induced insulin-resistant rats. Immunoblot analysis shows the expression of TNFα (**a**), TNFR1 (**b**), JNK (**c**), NF-ĸB (**d**), p-NF-ĸB p65 (**e**), COX2 (**f**), MCP1 (**g**), IL6 (**h**) and IL1β (**i**) in kidney tissues normalized to β-actin. Bar graphs show mean ± standard error of mean (SEM), *n* = 5–6 rats per group. ND: normal-diet-fed rats; HFD: high-fat-diet rats; HFD + AGOM: high-fat diet treated with agomelatine at a dose of 20 mg kg^−1^ day^−1^; HFD + PIO: high-fat diet treated with pioglitazone at a dose of 10 mg kg^−1^ day^−1^. * *p* < 0.05 vs. ND; † *p* < 0.05 vs. HFD.

The expressions of TNFα (Figure 2a) and TNFR1 (Figure 2b) were significantly increased in the HFD group in comparison to ND rats (*p* < 0.05). Similarly, the downstream signaling proteins, including JNK and p-NF-κB (p65 subunit), were significantly increased in HFD rats when compared with the ND group (*p* < 0.05) (Figure 2c,e). In HFD rats, the expression of NF-κB (p65 subunit) protein tended to increase when compared with ND (Figure 2d). The expression of the inflammatory cytokines COX2, MCP1, IL6 and IL1β was remarkably increased in HFD rats in comparison to the ND group (*p* < 0.05) (Figure 2f–i). AGOM significantly blocked the HFD-induced increases in TNFα, TNFR1, JNK, NF-κB and p-NF-κB (*p* < 0.05). Additionally, AGOM significantly inhibited the pro-inflammatory cytokines COX2 and IL1β (*p* < 0.05) (Figure 2f,h). However, the expression of IL6 tended to decrease but did not reach statistical significance compared to the HFD group. PIO demonstrated the potential to cause the inhibition of the release of COX2, IL6 and IL1β in comparison to HFD controls (*p* < 0.05) (Figure 2f,h,i). The data indicated that the obese condition aggravated damage to the kidney tissue through the stimulation of an inflammatory pathway, the TNFα/NF-ĸB cascade. These alterations were significantly ameliorated after treatment with AGOM and PIO. However, there was no significant difference between AGOM and PIO treatments.

### 2.5. Effects of Agomelatine on Kidney Necroptosis in High-Fat-Diet-Induced Obesity in Rats

**Figure 3:**
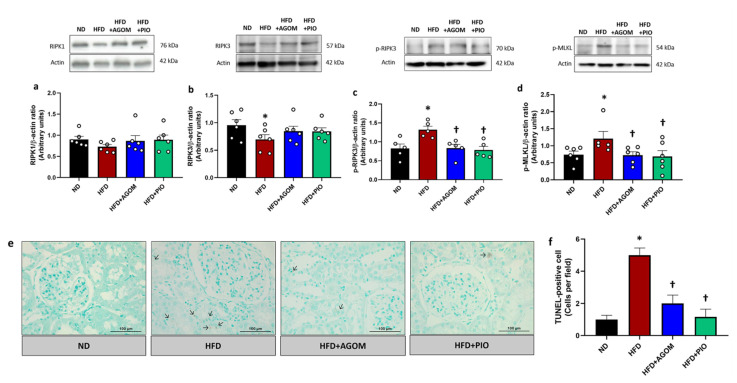
The effects of agomelatine on renal necroptosis signaling proteins in high-fat-diet-induced insulin-resistant rats. Immunoblot analysis shows the expression of RIPK1 (**a**), RIPK3 (**b**), p-RIPK3 (**c**) and p-MLKL (**d**) in kidney tissues normalized to β-actin. Renal cell death was represented by DNA fragmentation by using the TUNEL assay (**e**). The number of TUNEL-positive cells per field area (**f**). The back arrow indicates TUNEL-positive cells. Bar graphs show mean ± standard error of mean (SEM), *n* = 5–6 rats per group. ND: normal-diet-fed rats; HFD: high-fat-diet rats; HFD + AGOM: high-fat diet treated with agomelatine at a dose of 20 mg kg^−1^ day^−1^; HFD + PIO: high-fat diet treated with pioglitazone at a dose of 10 mg kg^−1^ day^−1^. * *p* < 0.05 vs. ND; † *p* < 0.05 vs. HFD.

The results demonstrated that there was no significant change in the expression of RIPK1 among the experimental groups (Figure 3a). The expression of RIPK3 in the kidneys of the HFD rats was significantly decreased in comparison to the ND controls (Figure 3b). The expression of both p-RIPK3 and p-MLKL in the kidney was stimulated significantly by the HFD condition in comparison to the ND group (*p* < 0.05) (Figure 3c,d). These results were consistent with the increase in apoptotic cells in the HFD group detected by the TUNEL assay (Figure 3e). AGOM or PIO treatment significantly suppressed the levels of the necroptosis markers, as shown by the decrease in the expression of p-RIPK3 and p-MLKL (Figure 3c,d). Moreover, the apoptotic cells in the kidney tissues were significantly attenuated in the AGOM and PIO-treated groups when compared with HFD rats (*p* < 0.05) (Figure 3e). These findings indicated that AGOM protected against renal cell death through the inhibition of the necroptosis pathway in this model.

### 2.6. Effects of Agomelatine on Impaired Insulin Signaling in High-Fat-Diet-Induced Obesity in Rats

**Figure 4:**
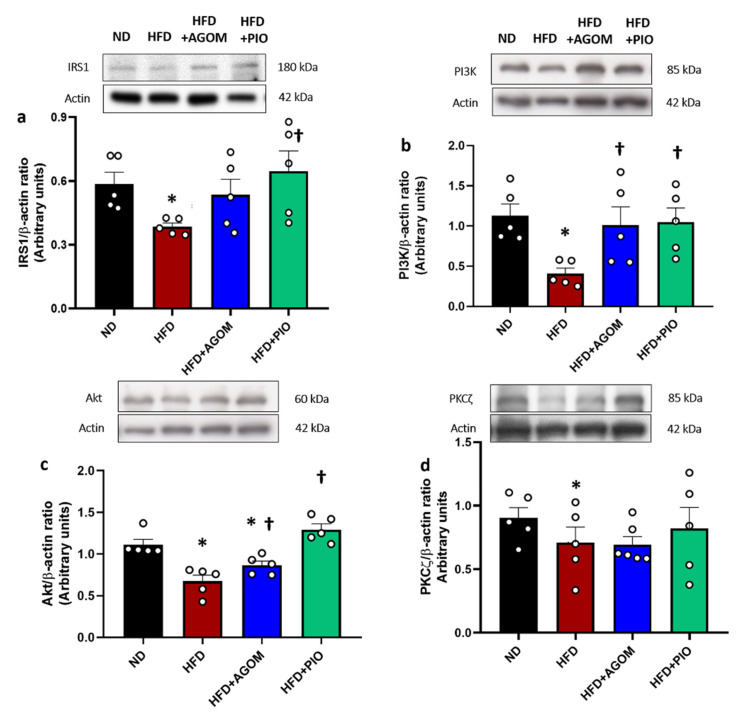
The effects of agomelatine on the renal insulin signaling pathway in high-fat-diet-induced insulin-resistant rats. Immunoblot analysis shows the expression of IRS1 (**a**), PI3K (**b**), Akt (**c**) and PKCζ (**d**) in kidney tissue normalized to β-actin. Bar graphs show mean ± standard error of mean (SEM), *n* = 5–6 rats per group. ND: normal-diet-fed rats; HFD: high-fat-diet rats; HFD + AGOM: high-fat diet treated with agomelatine at a dose of 20 mg kg^−1^ day^−1^; HFD + PIO: high-fat diet treated with pioglitazone at a dose of 10 mg kg^−1^ day^−1^. * *p* < 0.05 vs. ND; † *p* < 0.05 vs. HFD.

The expressions of IRS1, PI3K, Akt and PKCζ were significantly decreased in the HFD group when compared with ND rats (*p* < 0.05) (Figure 4a–d). The administration of AGOM improved insulin signaling, as demonstrated by the upregulation of the expression of PI3K and Akt in comparison to the HFD group (*p* < 0.05). The expressions of IRS1 and PKCζ were not significantly changed in AGOM when compared with the HFD controls. However, PIO significantly increased the IRS1 expression when compared with the HFD group (*p* < 0.05). These data indicated that renal insulin signaling was impaired in an obese condition. AGOM treatment could alleviate impaired insulin signaling in the kidney.

### 2.7. Effects of Agomelatine on Kidney Injury in High-Fat Diet-Induced Obesity in Rats

**Figure 5:**
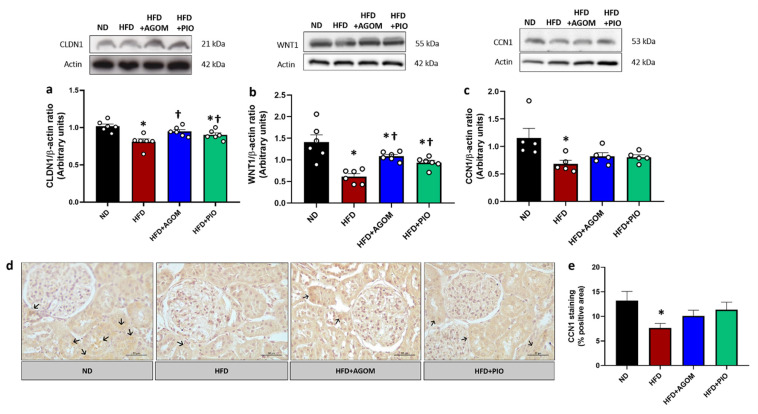
The effect of agomelatine on renal injury in high-fat-diet-induced insulin-resistant rats. Immunoblot analysis shows the expression of CLDN1 (**a**), WNT1 (**b**) and CCN1 (**c**) in kidney tissue normalized to β-actin. Immunohistochemistry (IHC) of CCN1 (**d**), the back arrow indicates the localization and expression of CCN1 and bar graphs % of positive area (**e**). Bar graphs show mean ± standard error of mean (SEM), *n* = 5–6 rats per group. ND: normal-diet-fed rats; HFD: high-fat-diet rats; HFD + AGOM: high-fat diet treated with agomelatine at a dose of 20 mg kg^−1^ day^−1^; HFD + PIO: high-fat diet treated with pioglitazone at a dose of 10 mg kg^−1^ day^−1^. * *p* < 0.05 vs. ND; † *p* < 0.05 vs. HFD.

The expression of CLDN1, WNT1 and CCN1 were significantly decreased in the HFD group when compared with ND rats (*p* < 0.05) (Figure 5a–c). The localization and expression of CCN1 in the obese conditions, which was verified by immunohistochemistry, was consistent with the western blot results (Figure 5d,e). Treatment with AGOM and PIO resulted in a significantly increased expression of CLDN1 and WNT1 when compared with the HFD controls (*p* < 0.05) (Figure 5a,b). AGOM or PIO treatment tended to increase CCN1 expression. These data indicated that AGOM treatment could prevent podocyte injury and activate cell proliferation and differentiation in an obese insulin-resistant condition.

## 3. Discussion

In this study, agomelatine was found to improve renal complications in rats with obesity induced by a high-fat diet. The results revealed that agomelatine has beneficial effects in protecting the kidneys by suppressing the expansion of adipose tissue, a major cause of systemic inflammation. The levels of expression of TNFα, TNFR1, NF-ĸB, p-NF-ĸB, and pro-inflammatory cytokines such as COX2 and IL-1β were reduced following agomelatine treatment. These effects were consistent with those observed following treatment with pioglitazone. Agomelatine is active via a novel mechanism that mitigates kidney injury through the inhibition of the TNFα/NF-ĸB/RIPK3 pathway. This action reduces inflammatory processes and necroptosis while also restoring the impaired renal insulin signaling pathway and conferring protection against kidney injury through the upregulation and recovery of glomerular tight junction proteins. Collectively, these effects contribute to protecting the kidneys against injury. This study demonstrated that agomelatine has more beneficial effects than pioglitazone, particularly with regard to the lowering of plasma cholesterol levels and the reducing of renal lipid accumulation.

Excess body weight can lead to several metabolic and physiological changes that negatively impact kidney function [2]. Our results indicated that the consequential impact of obesity was an increase in plasma cholesterol, a slight rise in plasma triglycerides, and an elevation of FFAs. These were associated with lipid accumulation in the kidneys of obese rats. This was evidenced by the upregulation of FAS and a modest increase in PLIN2 expression, which are markers of fatty acid synthesis. Lipid overload in kidney cells can lead to organelle dysfunction and the abnormal activation of intracellular signaling pathways, which is a condition known as lipotoxicity. Ren et al. reported that lipotoxicity plays a crucial role in acute kidney injury through several pathways, particularly via oxidative stress and ER stress [18]. Previous studies have demonstrated that FFAs in the blood are taken up into liver cells via the fatty acid transporter protein CD36. Then, FFAs are used to generate triglycerides through the action of FAS and accumulate as lipid droplets in the liver. Moreover, in ob/ob mice, elevated hepatic FAS activity subsequently resulted in the development of a fatty liver [19]. Therefore, the increase in serum FFAs in an obese condition attributed to elevated lipid synthesis and storage in renal tissues.

In this study, AGOM led to a modest reduction in serum FFAs, which in turn decreased fatty acid synthesis and lipid accumulation in the kidney. This was demonstrated by a reduction in FAS and a slight decrease in perilipin 2, a protein associated with lipid droplets. These changes suggested that AGOM may help shield the kidney from lipotoxicity. Interestingly, in terms of lipid metabolism, AGOM and PIO shared similar effects, including reductions in the visceral fat mass, adipocyte area, adipocyte size and plasma triglyceride levels. However, AGOM exerted greater efficacy in decreasing plasma cholesterol and increasing the adipocyte number than PIO treatment. Pioglitazone, a PPAR-γ agonist, drives the differentiation of preadipocytes into mature fat cells, increasing the number of cells dedicated to fat storage. This process enhances adipose tissue function and improves metabolic control, but does not directly reduce the adipocyte area [15].

It has been reported that treatment with AGOM alleviates VLDL production and hepatic lipid accumulation through the downregulation of FAS expression, which causes a decrease in fatty acid synthesis following fructose treatment in rats [20]. Additionally, supplementation with AGOM resulted in the attenuation of the lipid profile, which may be associated with the direct effect of the circadian rhythms that regulate dietary lipid absorption in intestinal enterocytes [21,22]. It also resulted in the modulation of the lipid metabolism and a reduction in plasma cholesterol, which were confirmed by our recent studies [5,6]. Therefore, it can be postulated that AGOM attenuated the accumulation of kidney lipids through the decrease in fatty acid synthesis in the kidney, thereby contributing to the mitigation of kidney injury.

We demonstrated that AGOM and PIO had similar effects regarding the reduction in renal inflammation in the obese condition. The anti-inflammatory effects of AGOM could be related to its ability to modulate anxiety and depression and reduce oxidative stress [23], which in turn can affect the expression of COX-2 and IL-1β. However, its specific impact on MCP-1 and IL-6 might be less pronounced due to the different signaling pathways involved in their expression. We propose that AGOM exhibited these effects by facilitating a decrease in adipocyte hypertrophy while instigating an increase in adipogenesis or adipocyte numbers, subsequently leading to a reduction in the generation of pro-inflammatory cytokines. Our findings are supported by a previous study demonstrating that AGOM treatment decreased the release of cytokines (TNF-α, IL-6 and IL-1β) and restored BDNF levels and the activity of the antioxidant enzymes CAT and GPx in the brain in an HFD-induced anxiety-like behavior model [23]. Moreover, a previous study reported that AGOM attenuated inflammation in the liver and adipose tissue cells of a HFD mice model through the inhibition of TNF-α gene expression, and led to a subsequent decrease in pro-inflammatory cytokines such as IL-1β, IL-6 and MCP-1 [22]. We purport that this inflammatory response can impair glomerular barriers and renal tubules. Our study demonstrated that serum creatinine, which is normally filtered out by the glomeruli and secreted by the renal tubules, remains in the blood circulation, indicating a reduction in glomerular and tubular function. Moreover, high levels of urine protein were observed in HFD rats. This change was alleviated by treatment with AGOM and PIO. These results confirmed that damage to the glomerular barriers occurs in obese rats and that AGOM and PIO could mitigate renal impairment.

We demonstrated for the first time that AGOM was highly effective in the prevention of renal necroptosis by blocking the TNFα/NF-ĸB/p-RIP3K signaling pathway, achieving results comparable to those of pioglitazone. We propose that the consumption of a high-fat diet results in the conversion of RIPK3 into its phosphorylated form, p-RIPK3. This phosphorylated protein then promotes the translocation of p-MLKL to the cell membrane. Once at the membrane, p-MLKL eventually undergoes degradation, which ultimately triggers renal cell death, as evidenced by the increase in DNA fragmentation evaluated by the TUNEL assay, a finding also supported by our recent study [5]. This cascade of events underscores the role of RIPK3 and p-MLKL in the pathology of renal cell injury associated with excessive dietary fat intake. AGOM and pioglitazone treatment evidently inhibited the expression of p-RIPK3 and p-MLKL and subsequently protected against kidney injury and DNA fragmentation in an obese condition. These findings were in alignment with the observation of reduced adipocyte hypertrophy and the decreased expression of TNF-α and NF-ĸB. Thus, AOGM and pioglitazone prevented kidney cell death and injury via the inactivation of the inflammatory pathway. which then attenuated renal cell necroptosis in this obese condition.

In this study, we demonstrated that insulin resistance was exhibited in the kidney following high-fat diet consumption. These changes made the kidneys unable to store glucose in the cells and provide nutrients to the cells, resulting in kidney cell damage. Recent studies have shown that renal insulin signaling is a key contributor and regulator of systemic glucose levels. These metabolisms were dysregulated under conditions of diabetes and insulin resistance [24,25,26]. It was found that the expression of NF-κB and JNK following the consumption of a high-fat diet disturbed insulin signaling proteins in the cells, resulting in insulin resistance [27]. Moreover, the dysregulation of PI3K/Akt causes several pathological diseases, including metabolic disease and diabetes mellitus [28]. Akt influences various metabolic processes in kidney cells, including glucose uptake and glycogen synthesis. This regulation of the carbohydrates is important for the maintenance of energy balance and overall cellular metabolism [29]. Our study demonstrated that treatment with AGOM and PIO showed comparable effects in improving the impaired insulin signaling pathway by enhancing the expression of PI3K and Akt. As AGOM and PIO have an essential role in the inhibition of inflammation in the kidneys, these drug treatments subsequently alleviated insulin resistance and restored insulin signaling in kidney cells. This result was supported by a previous study demonstrating that the anti-inflammatory activity of AGOM restored insulin signaling and effective glucose metabolism in an obese mice model [22]. AGOM also shows anti-oxidant properties, as it has been shown to protect against vascular dysfunction and kidney damage in an experimental model of high-fat-diet-induced insulin resistant rats [22].

In addition, we found that claudin-1 (CLDN1), a paracellular tight junction protein that plays a major role as a glomerular barrier and is highly expressed in podocytes, was obviously reduced in the kidney of obese rats. This alteration is linked to protein leakage from the glomerular barrier into the urine. The administration of AGOM and PIO significantly restored the expression of tight junction proteins and hence repaired the glomerular barrier, as evidenced by the reduction in urinary protein. However, the mechanisms responsible for the reduction in CLDN1 with the consumption of a high-fat diet are still unclear. We hypothesized that the impairment of the glomerular barrier might be due to inflammation and the stimulation of necroptosis-related proteins. Similar to previous studies, it was reported that renal inflammation contributed to a reduction in tight junction proteins in the kidney in a mouse model of necrotizing enterocolitis (NEC) [30]. Moreover, our previous study found that tight junction proteins were decreased under obese conditions, which were then related to renal oxidative stress and apoptosis. Treatment with AGOM at a dose of 20 and 40 mg kg^−1^ day^−1^ could upregulate the expression of these proteins [6]. This result was consistent with the decreased expression of WNT1 and CCN1 in the HFD rats in this study. CCN1 is primarily expressed in podocytes and is involved in cell adhesion, migration, and differentiation. In kidney disease, such as IgA nephropathy, diabetic nephropathy, and membranous nephropathy, CCN1 expression was significantly downregulated in podocytes, especially when there was substantial mesangial expansion [31]. We demonstrated that the reduction in CCN1 protein in the kidneys of HFD rats may affect podocyte cell differentiation, subsequently leading to glomerular barrier dysfunction. These results indicated that glomerular damage was observed in obese insulin resistant rats, as evidenced by protein leakage and high levels of serum creatinine. Inflammation and necroptosis could induce filtration barrier damage and inhibit renal proliferation, cell migration and differentiation, leading to kidney dysfunction. AGOM and PIO treatment protected against kidney injury in our model.

## 4. Materials and Methods

### 4.1. Animal and Experimental Design

Male Wistar rats at the age of 6–8 weeks, weighing 180–200 g, were obtained from Nomura Siam International, Bangkok, Thailand. The experiments were conducted in accordance with all approved procedures. All animal experiments were conducted at the Animal Facility, in accordance with the National Research Council’s Guide for the Care and Use of Laboratory Animals. The experimental procedures were authorized by the Institutional Animal Care and Use Committee of the Faculty of Medicine, Chiang Mai University, Chiang Mai, Thailand (Permit number: 03/2563).

The rats were housed in appropriate cages at a controlled temperature of 25 ± 1 °C, with a 12 h light/dark cycle, and were given ad libitum access to food and water. After acclimatization for 7 days, the rats were randomly assigned to two dietary groups: a normal diet (ND) group (*n* = 6) and a high-fat-diet (HFD) group (*n* = 18). The ND group were fed on standard rat chow (19.77% of energy from fat) and the HFD group on a HFD diet (59.28% of energy from fat) ad libitum for 16 weeks to induce the insulin-resistant condition, as described in our previous studies [5,6,32]. After week 16, animals in the HFD were subdivided into 3 groups: (1) HFD (*n* = 6), rats were given normal saline; (2) HFD + AGOM (*n* = 6) rats were given a high-fat diet plus agomelatine at a dose of 20 mg kg^−1^ day^−1^; and (3) HFD + PIO (*n* = 6) rats were given a high-fat diet plus pioglitazone at a dose of 10 mg kg^−1^ day^−1^. Both treatments were administered via oral gavage for 4 weeks. AGOM was purchased from Les Laboratoires (Les Laboratoires Servier, Gidy, France) and dissolved in normal saline solution. The dose of agomelatine 20 mg kg^−1^ day^−1^ was chosen with regard to its potential anti-inflammatory impact to avoid undesirable side effects. PIO, a positive control drug, was purchased from Berlin Pharmaceutical Industry Co., Ltd., Bangkok, Thailand. All rats were fed continuously with the HFD until week 20. The rats’ body weight and food intake were recorded once a week.

### 4.2. Animal Euthanasia, Tissue Harvest and Blood Collection

At the end of the study, the rats were euthanized via the administration of an overdose of isoflurane (at or above 3% *v*/*v* in air) until breathing ceased and reflexes were absent [33]. Blood, urine, adipose tissues and the kidneys were collected for further investigation. Once the rats were unconscious, the abdominal cavity was opened. Blood was drawn and collected from the abdominal aorta and kept in a tube containing either the anticoagulant, EDTA or sodium fluorides (NaF) or without an anticoagulant. The blood samples were centrifuged at 8500 rpm for 5 min, then serum or plasma were collected and stored at −20 °C until they were used for subsequent investigation.

The kidneys were removed, collected and placed in ice-cold phosphate-buffered saline (PBS); then, decapsulation was carried out immediately. One kidney was cut into a longitudinal section and subsequently fixed in 10% neutral-buffered formalin overnight for renal histological examination. The other half of the kidney was collected for further investigation. Tissues from the renal cortex were kept and placed at −80 °C until they were used for western blot analysis. Liver and visceral fat were removed from the abdominal cavity and kept at −80 °C for subsequent experiments.

### 4.3. The Measurement of Renal Function

At the end of week 20, the blood was collected. The serum creatinine level was determined using enzymatic colorimetric methods (DiaSys Diagnostic Systems GmbH, Holzheim, Germany). The data are presented as mg dL^−1^.

### 4.4. The Measurement of Urine Protein Levels

To evaluate renal function, the urine protein levels were evaluated. At the end of 20 weeks, 24 h urine samples were collected using metabolic cages. Immediately after collection, the urine samples were centrifuged at 10,000× *g* at 4 °C for 10 min. The supernatants from each sample were collected into clean tubes and stored at −20 °C until use. Before testing, the urine samples were diluted 1:50 with deionized water, and the concentration of urine protein was determined using a commercially available colorimetric Bradford protein assay kit (Bio-Rad, Philadelphia, PA, USA) at a wavelength of 595 nm. [6]. The protein concentration is expressed in mg mL^−1^.

### 4.5. The Measurement of Lipid Profiles and Lipid Droplet Accumulation

The plasma lipid profiles, including triglycerides and cholesterol, and serum FFAs, were determined. The serum FFA level was measured using a commercial Free Fatty Acid Quantitation Kit (MAK-044) (Millipore, MA, USA). The plasma triglyceride and cholesterol levels were measured using a commercial assay kit (Erba Mannheim, Mannheim, Germany). The plasma triglycerides and cholesterol were measured at 505 nm using a Bio-Rad iMark™ microplate absorbance reader (Bio-Rad Laboratories Inc., Hercules, CA, USA). The serum free fatty acid levels were measured at 570 nm using a BioTek Synergy™ microplate absorbance reader (BioTek, Winooski, VT, USA). The data pertinent to plasma triglyceride and cholesterol levels are presented as mg dL^−1^ and the serum free fatty acid data are presented as ng mL^−1^.

Visceral fat was obtained from the abdominal cavity. The fat tissues were fixed in 10% neutral-buffered formalin overnight, transferred into PBS on the next day and left to soak in PBS until being embedded in paraffin. Then, 2 μm thick sections of paraffin-embedded specimens were stained with hematoxylin and eosin (H&E) for general histological assessment. The slide samples were visualized to show lipid droplets in the adipose tissue and were viewed under a light microscope (bright-field) (Leica DM 750) (Leica Microsystems, Wetzlar, Germany). The adipocyte area (μm^2^ × 10^3^), adipocyte size (μm) and adipocyte number (cells per field of view) of the lipid droplets were analyzed using Leica Application Suite (LAS) software version 4.12.

### 4.6. The Measurement of Plasma Insulin and HOMA-IR

Commercial kits were used to detect the concentration of plasma insulin in accordance with the manufacturers’ instructions. The plasma insulin levels were determined using an ELISA kit (EZRMI-13K) (Millipore, MA, USA). Fasting glucose was determined using commercial assay kits (Erba Mannheim, Mannheim, Germany). The homeostasis model assessment (HOMA) index was calculated as follows: HOMA-IR = (Fasting insulin level (ng mL^−1^) × Fasting glucose level (mg dL^−1^))/405.

### 4.7. Protein Detection and Western Blot Analysis

After euthanasia, the abdominal cavity was opened. The kidneys were removed and placed on ice-cold PBS. Then, each kidney was cut longitudinally, and the renal cortex was separated. Tissue from the renal cortex was homogenized and centrifuged at 5000× *g* for 10 min at 4 °C and then the supernatant was collected and designated as the whole cell lysate fraction. After this earlier step, this fraction was centrifuged continuously at 100,000× *g* for 2 h at 4 °C. The supernatant from this step was used as the cytosolic fraction and the pellet from this step was used to equate to the cell membrane fraction. The pellet from the whole cell lysate extraction step was resuspended and centrifuged at 10,000× *g* at 4 °C for 10 min, then the supernatant was collected as a nuclear fraction. All fractions were kept at −80 °C pending further investigations. The protein concentration was detected using a colorimetric Bradford protein assay (Bio-Rad, PA, USA). An 80 μg sample of tissue proteins was used for western blot analysis. The proteins were separated on 8–12% gel by SDS-PAGE, a procedure that followed the electrophoresis methods described in our previous study [6]. After the gel running process, the proteins in the gels were transferred at 100 volts onto a polyvinylidene fluoride membrane (PVDF) (GE Healthcare, Buckinghamshire, UK) for 60–75 min. Then, the membranes were soaked in Ponceau S solution (Sigma-Aldrich, St. Louis, MO, USA) to stain the proteins on the PVDF membranes. The PVDF membranes were cut according to the molecular weight of proteins and then these membranes were incubated and blocked with 2–5% non-fat milk for 60 min at room temperature. After 60 min of incubation with non-fat dry milk, the membranes were incubated with primary antibodies. Primary antibodies including perilipin-2 (PLIN2), claudin-1 (CLDN1) (Abcam, MA, USA), FAS, TNFR1, NF-κB (p65), p-NF-κB (p65), lamin-1, actin, IRS1, PI3K, PKCζ, COX2 (Cell Signaling Technology, Danvers, MA, USA), TNFα, RIPK1, Akt, CCN1, IL-1β (Millipore, MA, USA), RIPK3, p-RIPK3, p-MLKL, WNT1 (Invitrogen, Waltham, MA, USA), and IL-6 (Santa Cruz Biotechnology, Santa Cruz, CA, USA) were used. The membranes were continuously incubated with the specific horseradish peroxidase (HRP)-conjugated goat anti-rabbit IgG secondary antibody (Millipore, MA, USA). An enhanced chemiluminescence agent (Bio-Rad Laboratories Ltd., Hercules, CA, USA) was used for the detection of the immunoreactive bands on the membrane. The membranes were then investigated using the reactive signal and a gel documentation system (OMEGA LUM^TM^ G Imaging System) (Aplegen, Inc., Pleasanton, CA, USA) or the membranes were exposed to a Hyperfilm (Hyperfilm ECL, GE Healthcare, Buckinghamshire, UK) in an X-ray film cassette. The densities of the bands were subsequently analyzed using ImageJ software version 1.52a and all densities of the bands were normalized with β-actin. The data are presented as arbitrary units.

### 4.8. The Measurement of Renal Cell Apoptosis or DNA Fragmentation by TUNEL Assay

DNA fragmentation was investigated using Terminal deoxynucleotidyl transferase dUTP nick end labeling (TUNEL assay) using a TdT-FragEL^TM^ DNA fragmentation detection kit, in accordance with the manufacturer’s instructions (Millipore, Billerica, MA, USA). The fragmentation was detected from the attachment between a modified dUTP and the 3′-OH end of the damaged DNA using the enzyme terminal deoxynucleotidyl transferase dUTP nick end labeling (TUNEL) reaction. Six fields in each section were randomly selected for analysis and reported as the average TUNEL-positive cells/field, as described in our previous study [5,7].

### 4.9. Immunohistochemical Study

Firstly, the unstained kidney slides were deparaffinized using xylene and rehydrated with a series of ethanols. Subsequently, these slides were incubated with 3% H_2_O_2_ for 30 min to block endogenous peroxidase activity. Then, the slides were incubated overnight at 4 °C with the CCN1 primary antibody (Millipore, MA, USA) at a concentration of 1:200. After washing, the slides were incubated with Biotinylated Secondary Antibody (Goat Anti-Rabbit IgG). A detection kit was used, in accordance with the manufacturer’s instructions, to determine and detect the positive area in each sample under light microscopy (bright-field) (Leica DM 750) (Leica Microsystems, Wetzlar, Germany). Six fields on each kidney slide were randomly selected to calculate the total number of positive areas. The data are presented as the percentage of positive areas.

### 4.10. Statistical Analysis

Bar graphs and dot plots were produced by GraphPad Prism version 8. To compare the data between groups, we used IBM SPSS Statistics version 22. A one-way ANOVA followed by Fisher’s least significant difference test (LSD) was used for all data. All data are expressed as mean ± standard error of mean (SEM), with statistical significance determined when the *p* value is less than 0.05.

## 5. Conclusions

The novel finding of this study is that agomelatine, to a similar extent to pioglitazone, mitigates kidney damage in obese insulin-resistant rats by inhibiting inflammation and necroptosis through the TNF-α/NF-ĸB/p-RIPK3 pathway and by improving insulin signaling. AGOM demonstrated greater cholesterol-lowering effects than pioglitazone. However, AGOM and pioglitazone had similar levels of efficacy in reducing renal inflammation and restoring impaired renal function in this model. Our results may pave the way for the use of AGOM in the prevention of kidney injury and dysfunction in the case of prediabetic or obese conditions. However, the molecular mechanisms concerning these actions need to be further elucidated.

## Figures and Tables

**Table 1:** Effects of high-fat diet feeding for 16 weeks (before treatment) on metabolic parameters and renal function in rats.

Parameters	ND	HFD	HFD + AGOM	HFD + PIO
Initial body weight (g)	206.7 ± 2.10	210.8 ± 2.71	207.5 ± 3.35	204.2 ± 2.71
Body weight (g)	538.33 ± 16.0	685.0 ± 30.0 *	614.0 ± 23.57 *	615.0 ± 12.31 *
Fasting plasma glucose (mg dL^−1^)	76.19 ± 8.68	111.98 ± 7.82 *	104.92 ± 8.03 *	101.25 ± 9.22 *
Plasma insulin (ng mL^−1^)	3.86 ± 0.05	5.67 ± 0.72	5.57 ± 0.63	5.19 ± 0.74
HOMA-IR	0.87 ± 0.14	1.69 ± 0.34 *	1.65 ± 0.28 *	1.46 ± 0.13
Serum creatinine (mg dL^−1^)	0.52 ± 0.01	0.56 ± 0.01 *	0.55 ± 0.01 *	0.56 ± 0.01 *
Plasma triglycerides (mg dL^−1^)	46.26 ± 7.88	52.31 ± 5.30	56.58 ± 6.61	52.69 ± 2.99
Plasma cholesterol (mg dL^−1^)	72.02 ± 4.33	77.61 ± 6.93	74.74 ± 3.66	77.36 ± 14.29

Notes: The effects of consumption of a high-fat diet on metabolic parameters and renal function. The data show mean ± standard error of mean (SEM), *n* = 5–6 rats per group. ND: normal-diet-fed rats; HFD: high-fat diet rats; HFD + AGOM: high-fat diet treated with agomelatine; HFD + PIO: high-fat diet treated with pioglitazone. * *p* < 0.05 vs. ND.

**Table 2:** Effect of agomelatine on metabolic parameters, insulin resistance, kidney function and lipid profile in high-fat-diet-induced insulin-resistant rats at the end of the study treatment (week 20).

Parameters	ND	HFD	HFD + AGOM	HFD + PIO
Body weight (g)	555 ± 15.97	793 ± 33.00 *	631 ± 20.14 *^†^	711 ± 27.47 *^†‡^
Kidney weight (g)	1.41 ± 0.06	1.61 ± 0.09	1.28 ± 0.05 ^†^	1.35 ± 0.04 ^†^
Kidney/body weight ratio	0.25 ± 0.005	0.20 ± 0.003 *	0.20 ± 0.006 *	0.19 ± 0.005 *
Fasting plasma glucose (mg dL^−1^)	76.81 ± 2.62	108.90 ± 2.04 *	95.96 ± 4.23 *^†^	89.20 ± 6.02 *^†^
Plasma insulin (ng mL^−1^)	3.80 ± 0.46	9.31 ± 2.04 *	5.73 ± 0.66 ^†^	4.79 ± 0.93 ^†^
HOMA-IR	0.64 ± 0.09	2.40 ± 0.62 *	1.16 ± 0.28 ^†^	0.88 ± 0.15 ^†^
Serum creatinine (mg dL^−1^)	0.54 ± 0.01	0.59 ± 0.01 *	0.56 ± 0.01 ^†^	0.56 ± 0.02 ^†^
Urine protein (mg mL^−1^)	1.14 ± 0.05	1.77 ± 0.04 *	1.05 ± 0.01 ^†^	1.14 ± 0.06 ^†^
Serum free fatty acids (nmole ul^−1^)	0.29 ± 0.04	0.49 ± 0.08 *	0.34 ± 0.04	0.31 ± 0.78
Plasma triglycerides (mg dL^−1^)	51.53 ± 5.44	58.93 ± 6.78	54.79 ± 3.50	52.03 ± 5.20
Plasma cholesterol (mg dL^−1^)	50.15 ± 2.10	86.76 ± 6.34 *	52.82 ± 1.88 ^†^	74.63 ± 10.98 *

Notes: The effects of agomelatine and pioglitazone treatments on metabolic parameters, insulin resistance, kidney function, and lipid profile in high-fat-diet-induced insulin resistant rats. The data show mean ± standard error of mean (SEM), *n* = 5–6 rats per group. ND: normal-diet-fed rats; HFD: high-fat diet rats; HFD + AGOM: high-fat diet treated with agomelatine at the dose of 20 mg kg^−1^ day^−1^; HFD + PIO: high-fat diet treated with pioglitazone at the dose of 10 mg kg^−1^ day^−1^. * *p* < 0.05 vs. ND; † *p* < 0.05 vs. HFD; ‡ *p* < 0.05 vs. AGOM.

## Data Availability

The datasets generated and/or analyzed during the current study are available from the corresponding author upon reasonable request.
